# Microstructure, Mechanical Properties and Fatigue Crack Growth Behavior of Gas Tungsten Arc Welding Welded Joint of the Hastelloy N Alloy

**DOI:** 10.3390/ma16196510

**Published:** 2023-09-30

**Authors:** Sai Wang, Baoyun Ma, Daochen Feng, Shuangjian Chen, Yinghe Ma, Huaxin Li, Chuanyang Lv, Wenjian Zheng, Jianguo Yang

**Affiliations:** 1 College of Mechanical Engineering, Zhejiang University of Technology, Hangzhou 310014, China; wangsai12226@126.com (S.W.); 19550282851@163.com (B.M.); fengdc@zjut.edu.cn (D.F.); mayh@zjut.edu.cn (Y.M.); hxli2019@zjut.edu.cn (H.L.); cylv@zjut.edu.cn (C.L.); 2 Institute of Process Equipment and Control Engineering, Zhejiang University of Technology, Hangzhou 310014, China; 3 Shanghai Institute of Optics and Fine Mechanics, Chinese Academy of Sciences, Shanghai 201800, China; chenshuangjian@siom.ac.cn; 4 Institute of Innovation Research, Shengzhou Zhejiang University of Technology, Shengzhou 312400, China

**Keywords:** Hastelloy N alloy, welding, mechanical property, fatigue performance

## Abstract

Hastelloy N alloy is an excellent oxidation and corrosion-resistant material, which is selected as the shell material for the main vessel of molten salt reactors (MSRs). In this work, we conducted double-sided gas tungsten arc welding (GTAW) on 4 mm thick Hastelloy N alloy plates to examine the microstructure and mechanical properties of the welded joints. The S−N curve was obtained by fatigue test. The experimental results show that fatigue cracks initiate along the weld toe and propagate inward in a fan-shaped pattern. The hardness is highest in the heat-affected zone (HAZ). The fracture mode observed was trans-granular. The plastic zone in the initial stages of crack propagation remained relatively minimal. However, it gradually expanded during subsequent stages of the process. It is noteworthy that the crack propagation process often involves the development of secondary cracks, accompanied by profound plasticity-induced closure effects. The results of our investigation demonstrate that the welded joint exhibits excellent fatigue performance.

## 1. Introduction

Since the Fukushima nuclear accident in Japan, molten salt reactors have gained significant popularity among Generation IV nuclear reactors due to their remarkable reliability, safety, and sustainability [1,2,3,4]. The Oak Ridge National Laboratory, the research institute for the development of MSRs, has designed Hastelloy N alloy for the main vessel shell materials of MSRs [5,6]. Extensive systematic studies have demonstrated that this material offers exceptional resistance to oxidation and corrosion, making it well-suited to withstand the severe high-temperature corrosion conditions prevalent in MSRs [7].

In the current research, the focus on welding of Hastelloy N alloy primarily revolves around weldability and reheat crack sensitivity [8,9,10,11,12,13,14,15]. Wang W. et al. [16] have studied the fillet tungsten inert gas welding conditions of Hastelloy N alloy welded joints, particularly analyzing the influence of Si alloying element on carbide morphology. Chen S. et al. [17,18] have shown that the carbides present in the heat-affected zone (HAZ) and weld seam are M_6_C carbides. Furthermore, the welded joints exhibit good mechanical properties between 650 °C and 725 °C, and the plastic strain hardening behavior in the HAZ leads to the joint’s tensile fracture occurring in the base material (BM) region. Yu K. et al. [19,20] characterized the microstructure of domestically produced Hastelloy N (GH3535) alloy laser-welded joints and examined the evolution of their heat treatment process along with the mechanical properties of the welded joints. He Y. et al. [21] optimized the microstructure and mechanical properties of Hastelloy N high-temperature alloy joints by adjusting brazing process parameters and thermal exposure. Their approach involved removing borides from the isothermal solidification zone and increasing carbides in this region, leading to enhanced strength of the brazed joint. Moreover, several scholars have focused on evaluating the resistance of Hastelloy N alloy welded joints to molten salt corrosion and radiation [22,23,24,25,26].

During the operation of the MSRs, the main vessel of the reactor experiences pressure fluctuations, which can significantly impact the structural integrity. Welded joints and the overall fatigue damage of the welded structure are particularly vulnerable in this cyclic loading scenario. However, there is a lack of comprehensive research focusing on the fatigue behavior of alloy welded joints. Given the importance of maintaining the integrity of the welded structure of the MSRs main vessel under alternating load conditions, along with the presence of significant tissue gradients, stress gradients, and performance gradients in the welded joint, it is crucial to investigate the fatigue damage mechanism of Hastelloy N alloy welded joints.

By investigating the organization morphology, mechanical properties, and fatigue damage mechanism of Hastelloy N alloy welded joints, the exploration of the fatigue damage mechanism in Hastelloy N alloy welded joints fills a critical knowledge gap in the current literature. The findings from this study not only deepen our understanding of the underlying fatigue mechanisms but also provide valuable guidance for ensuring the integrity of welded structures in the main vessel of MSRs during operation.

## 2. Materials and Methods

**Figure 1:**
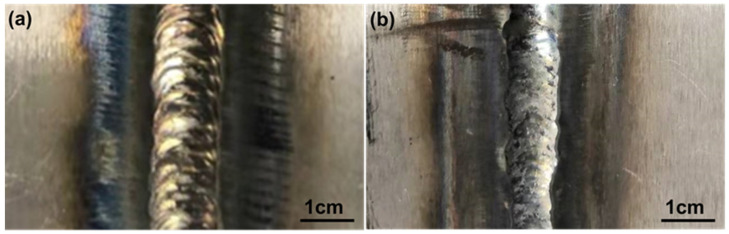
The macro morphology of GTAW double-sided welding joint of Hastelloy N alloy: (**a**) face weld; (**b**) back weld.

Hastelloy N alloy plates with a thickness of 4 mm were used for GTAW. The welding wire utilized is Φ 2.4 mm ERNiMo−2 wire. The groove, cut along the length of the plate, features a bevel angle of 30°. The root gap of 1mm is maintained. The fixture is used to locate and clamp the BM in the way of line compression during the welding test to ensure the welding quality and reduce the welding deformation. Grind and remove the surface oxide layer before welding, and clean the test piece. After completing the front welding, remove the weld root and perform back welding. High-purity argon (99.99%) was used as welding-shielded gas during the whole welding process. Details regarding the welding process specifications can be found in Table 1. The surface morphology of the welded joint is shown in Figure 1. It indicates the visually appealing appearance of the weld with no discernible defects. Subsequently, an X-ray inspection was conducted, revealing the absence of any weld defects.

**Figure 2:**
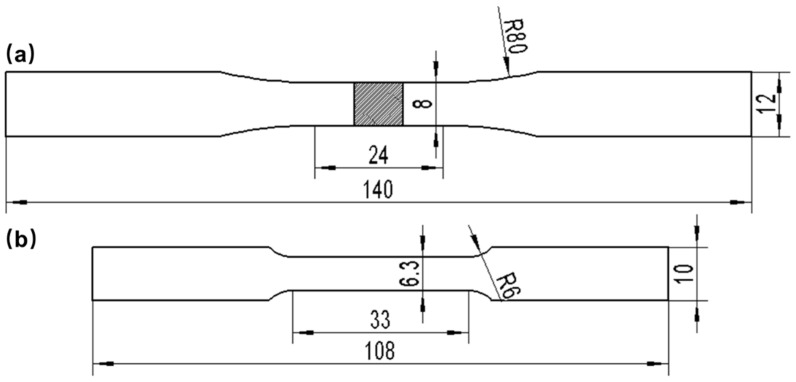
Dimension of the sample for (**a**) tensile specimens and (**b**) fatigue specimens.

**Figure 3:**
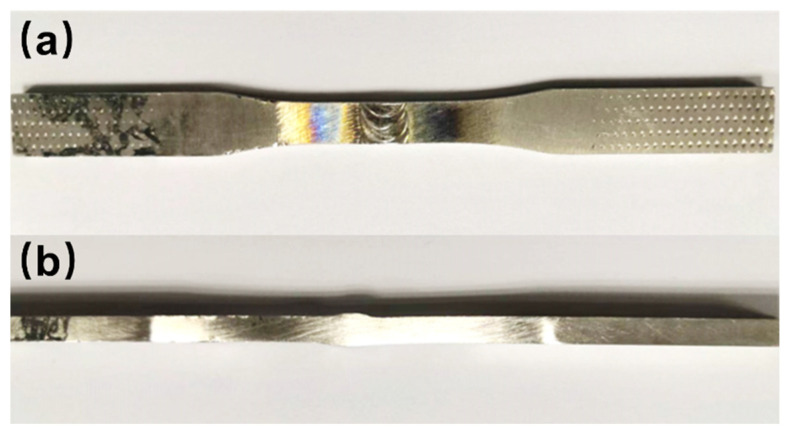
Macroscopic photographs: (**a**) front; (**b**) cross−sectional.

In the experiment, the specimens were electrical discharge wire-cut, ground, polished, and then etched with a solution of 20 mL H_2_O, 10 mL HCl, and 1.2 g FeCl_3_ for 60 s at room temperature. Microstructure characterization and analysis were conducted using a scanning electron microscope (SEM) (Quanta 200F, FEI, Holland) coupled with an energy-dispersive X-ray spectroscopy (EDS) (Nano X Flash Detector 5010, Bruker, Germany). The ZEISS LEO 1530VP SEM, equipped with an Oxford electron back-scattered diffraction (EBSD)(), was used to acquire information on grain boundary characteristics and local strain distribution. Hardness tests were conducted using a ZHV 30 micro Vickers hardness tester, applying a loading load of 500 gf and a holding time of 15 s. Tensile and fatigue tests were carried out in accordance with ASTM E8 and ASTM E466 standards, respectively. Please refer to Figure 2 for information on specimen dimensions and shape. Macroscopic photographs of the specimens are shown in Figure 3. The tensile properties of specimens were tested by an Instron 3369 universal material machine, and stress-strain curves were measured at a loading rate of 3 mm/min. For the stress-controlled fatigue loading tests, a sinusoidal uniaxial cyclic loading was applied at a frequency of 10 Hz and a loading rate of R = 0.1. Fatigue tests performed on welded joints of Hastelloy N alloy were conducted under various external maximum stresses ranging from 250 MPa to 290 MPa (in the elastic). All experiments were performed at room temperature.

## 3. Results and Discussion

### 3.1. Microstructure

**Figure 4:**
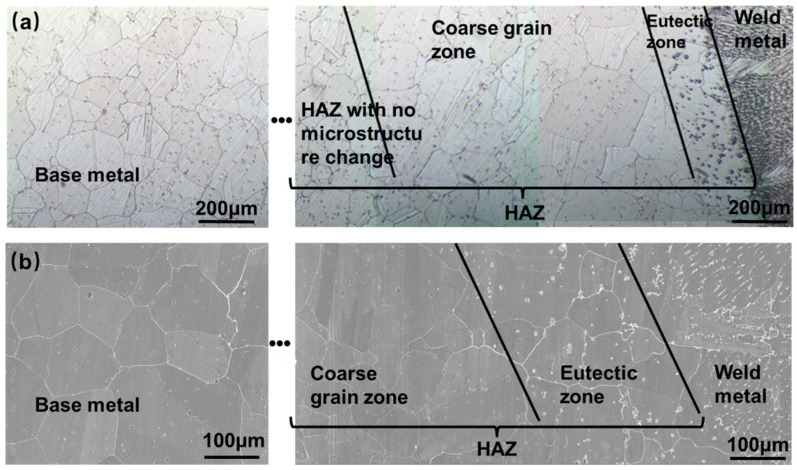
Images of welded joint (**a**) OM; (**b**) SEM.

Optical metallographic (OM) and SEM photographs of the cross-section of the welded joint in the welding state of Hastelloy N alloy are shown in Figure 4. It can be seen that the joint is divided into three different zones, namely the BM, the HAZ, and weld metal (WM). The BM is composed of a single austenite tissue, with fine carbides diffusely distributed on the matrix. The HAZ can be subdivided into three different zones, namely the eutectic transformation zone of precipitates (ETZ), the coarse grain zone (CGZ), and the critical zone with no significant microstructure changes (CZ). The ETZ is located near the fusion line and has a width of about 250 μm, where large amounts of precipitates are evident. The width of the CGZ is approximately 1000 μm, and the grain size in this zone has grown slightly compared to the left side of the zone. The left side of the HAZ is a zone where no significant tissue changes can be seen from the metallographic photograph, and the width of this zone cannot be accurately determined from the metallographic photograph alone. In the WM, a large number of columnar crystals can be seen at almost perpendicular angles to the fusion line, and a large number of precipitates exist within the columnar crystals and at the grain boundaries.

**Figure 5:**
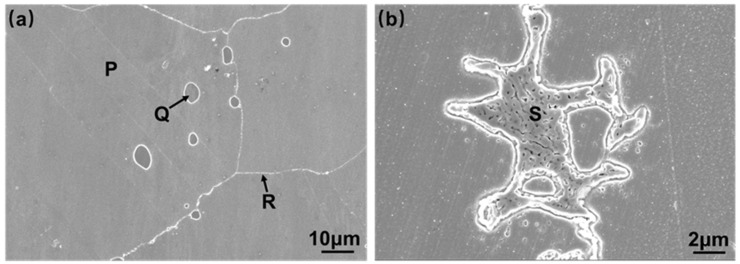
EDS analysis of the phases in Hastelloy N alloy welded joint: (**a**) base material; (**b**) spheroidized carbides.

Figure 5 shows the SEM photographs of the local magnification of the BM region and fusion line region of the welded joint. Figure 5b shows that the eutectic structure in ETZ undergoes spheroidization under the action of the welding heat source while still maintaining the transformed lamellar morphology. According to the EDS composition analysis results in Table 2, phase P is a Ni-based solid solution. Combined with the relevant literature reports [21,27], the gray phase Q and S are speculated to be M_6_C. M_6_C usually exists in three forms: A_3_B_3_C, A_4_B_2_C, and A_2_B_4_C, where the phase P represents Co, Cr, Fe, Mn, Ni, and V, and the phase Q represents Mo, Nb, Ta, Ti, V, W, and Zr [28]. In this study, phase P represents Ni, Cr, and Fe elements, and the phase Q represents Mo and Ti elements. Si atoms can occupy the Mo(16d) atomic position in the M_6_C structure, leading to a decrease in M_6_C lattice parameters and a more stable M_6_C structure. Under high-temperature conditions, Si-doped M_6_C is easier to precipitate and more stable than M_6_C without Si [28].

**Figure 6:**
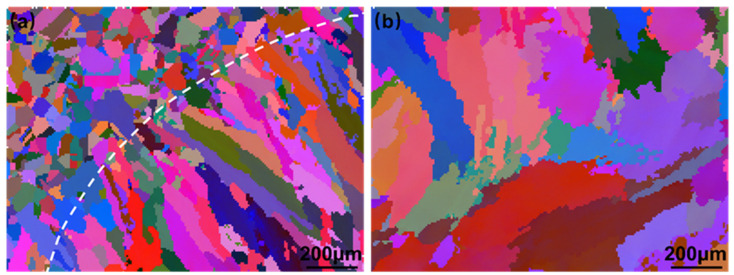
Grain morphology of the fusion zone by EBSD analysis: (**a**) fusing line area; (**b**) melting pool center.

Figure 6 shows the grain morphology distinguished by different colors using the EBSD technique, from which a high-angle solidification grain boundary with a difference in phase greater than 15° can be observed. At the fusion line, the grain solidification growth mode was found to be epitaxial growth. The fusion line at the existing grains acted as the nucleation of the matrix in the welding process, as the melt pool liquid metal was able to completely wet the matrix grains, allowing the crystal to be easily nucleated on the matrix grains without changing the existing grain orientation. Based on the solidification theory of the weld pool [29], the temperature gradient *G* near the fusion line was steeper than that in the center of the weld pool, but its growth rate *R* was small. The ratio of *G/R* gradually decreased from the fusion line to the inner part of the weld pool. This indicated that the degree of undercooling of its composition gradually increased from the fusion line to the center of the weld pool, resulting in a solidification mode transition from columnar dendritic crystals to cellular crystals and then to equiaxed crystals in the center.

### 3.2. Hardness Distribution

**Figure 7:**
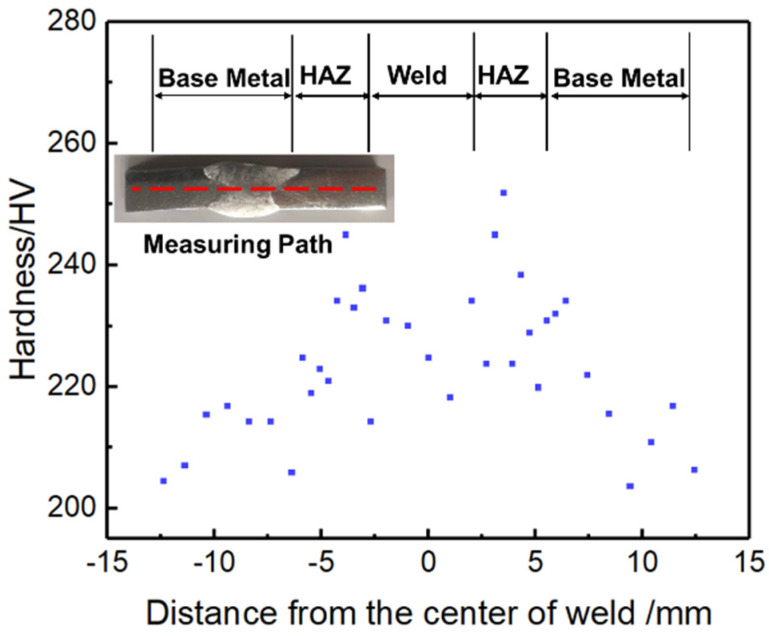
Microhardness distributions of the cross−section of the joint. (The red dotted line is the measurement path, and the blue dot is the hardness value at the corresponding location.)

The hardness distribution along the centerline of the welded joint cross-section is shown in Figure 7. It is observed that the weld metal exhibits symmetrical behavior, characterized by the presence of maximum hardness values on both sides of the fusion line. These values exceed 250 HV at this location. The average hardness of the WM is lower than that of the HAZ, which is distributed in the range of 210 to 250 HV and gradually decreases as it moves away from the fusion line. The BM is the lowest hardness area in the whole joint, with hardness values mostly below 220 HV. The hardness of a material is mainly derived from dislocation density, solid solution hardening, and precipitation hardening [30]. Nickel-based high-temperature alloys contain a significant amount of carbide-forming elements, such as Cr, Mo, and Ti. These elements determine the alloy’s microstructure and complex phase compositions, which contribute to its solid solution strengthening and precipitation hardening [31]. The presence of more carbides in the weld and heat-affected zone plays a vital role in enhancing the alloy’s strength. Observations from Figure 4 reveal abundant precipitates in the ETZ. These precipitates not only impede dislocation movement within the crystals but also strengthen the grain boundaries. As a result, the hardness of the alloy increases.

### 3.3. Tensile Properties

**Figure 8:**
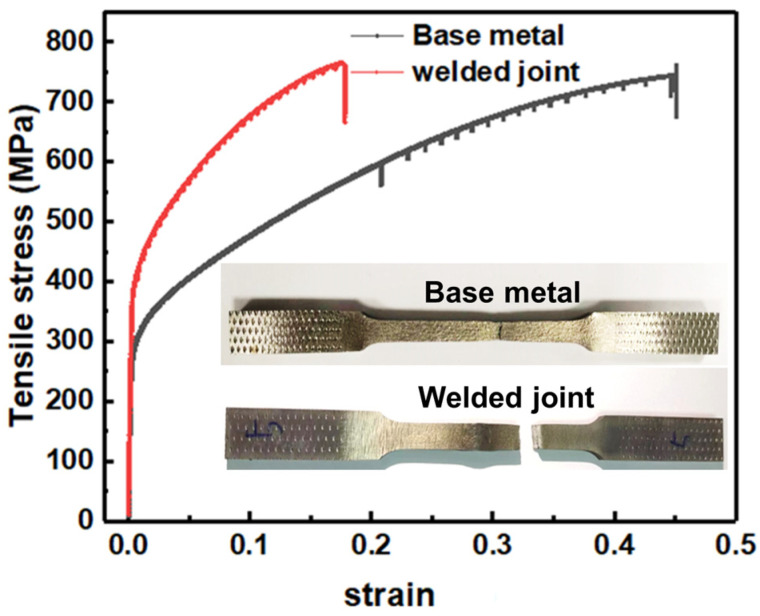
Stress versus strain curves of BM and welded joint.

Figure 8 shows the stress-strain curve for the BM and welded joints at room temperature. It is clear that the weld strength is increased, its elongation is decreased, the yield strength of the BM and welded joints were 290 MPa and 380 MPa, respectively, and the tensile strength is relatively close, 760 MPa and 764 MPa, as shown in Table 3. Fracture occurred in the BM area, the strength of the weld and heat-affected zone is higher compared to the BM, the BM is the weak link in the performance of the welded joint. The necking phenomenon exists near the fracture area because of plastic deformation. Deformation in materials arises from crystal slip and grain boundary slip, which are attributed to the inherent crystal structure. These slip processes are activated by applied stress, leading to stress concentrations and localized deformation. Consequently, sawtooth features are formed. The HAZ experiences thermal cycling and phase transformation, along with grain growth, recrystallization, and the development of residual stresses. As a result, the dislocation density in the HAZ is observed to be higher than that in the BM. The HAZ undergoes local plastic deformation due to the welding heat source, resulting in a significantly higher dislocation density than that of the BM. This leads to increased strength and reduced plastic deformation susceptibility within the HAZ. The excellent properties in the weld zone are mainly influenced by the precipitation of M_6_C-_γ_ eutectic phase. Although the weld segregation during solidification may weaken the effect of solid solution strengthening in the weld zone, the precipitation of M_6_C-_γ_ eutectic between the dendrites and on the solidification grain boundaries can compensate for the strength weakening. This mechanism can be explained by the Orowan mechanism [32].

### 3.4. Fatigue

**Figure 9:**
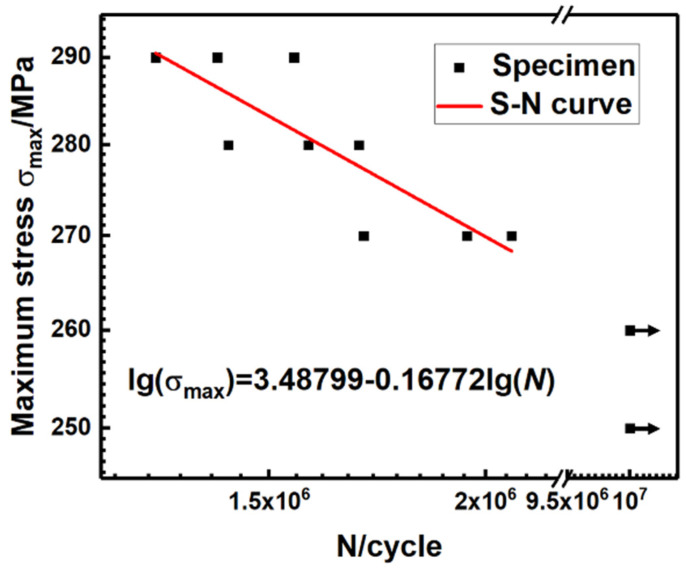
S−N curve of Hastelloy N alloy welded joint.

The S−N curves shown in Figure 9 show the fatigue performance of Hastelloy N alloy joints in high-cycle fatigue tests. By fitting the data using the least squares method, all data are expressed on a double logarithmic scale, and the regression line in Figure 9 represents the S−N curve at a 50% probability of survival. On the S−N curve, the welded joint shows a shorter fatigue life at the maximum stress of 270−290 MPa and shows a fatigue strength of 260 MPa, which can be considered as the fatigue limit (based on 10^7^ cycles). It is noted that although the maximum stress is close to the yield strength, the material still has a high fatigue life of 1.29 × 10^6^ cycles, which is related to the lower strength-to-yield ratio and superior material plasticity. The S−N curve of this welded joint can be approximated by the following equation.

(1) $$\mathrm{lg}\left(\sigma_{max}\right)=3.48799-0.16772\mathrm{lg}\;(N)$$

where *N* is the number of cycles to failure, *σ_max_* is the maximum stress. Equation (1) is based only on the data corresponding to the fracture specimens.

**Figure 10:**
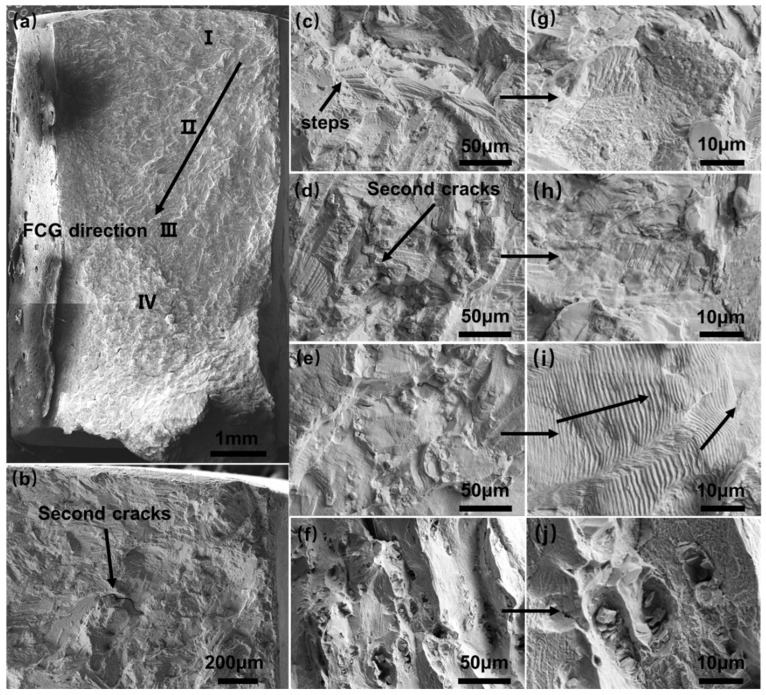
SEM of images impact fracture of Hastelloy N alloy welded joint: (**a**) overall morphology; (**b**) crack source; (**c**) local area of region I; (**d**) local area of region II; (**e**) local area of region III; (**f**) local area of region IV; (**g**) local magnification of (**c**) in the figure; (**h**) local magnification of (**d**) in the figure; (**i**) local magnification of (**e**) in the figure; (**j**) local magnification of (**f**) in the figure.

Under cyclic fatigue loading, the dislocation slip and repeated plastic deformation cause the small micro-cracks to extend continuously and connect to the macro fatigue crack. The fatigue crack propagation (FCG) leaves certain distinguishable characteristics on the final fracture. As illustrated in Figure 10 for the welded joint under the maximum stress of 270 MPa, the fatigue fracture morphology can be divided into four areas: the fatigue source area, the stable extension area, the fast-spreading area, and the transient breaking area. Cracks emerge from the corners and fan out diagonally, as seen in Figure 10b,c,g of the fatigue source area. Here, the fatigue striations on the fracture surface are barely visible, and secondary cracks and small steps are present. Figure 10d,h of the stable expansion area shows similar features, with secondary cracks and more distinct fatigue striations. Figure 10e,i of the rapid expansion area reveals even clearer fatigue striations, with increasing spacing and fewer secondary cracks. Planes of varying sizes, delineated by tear edges, intersect at an angle, indicating that the crack has deviated during its expansion. This phenomenon can be attributed to the varying orientations of adjacent grains, grain boundaries, or hard phases in relation to the crack tip. These factors impede the crack’s growth and induce a shift in its direction, causing it to expand along the path of least resistance. Figure 10f,j show transient fault fracture morphology, fatigue crack length increasing high-speed expansion at this stage, after reaching the critical length of crack instability of expanding quickly and fracture. The fracture surface exhibits mixed characteristics, with shallow dimples and fragments formed by ductile fracture. The microstructure mainly shows instantaneous fracture features, except for the small amount of fatigue striations observed locally.

**Figure 11:**
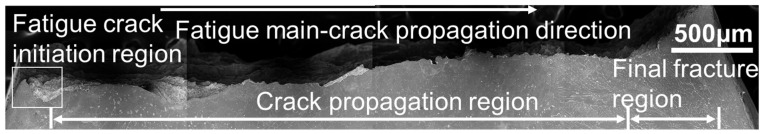
Organizational characteristics of fatigue fracture profile.

**Figure 12:**
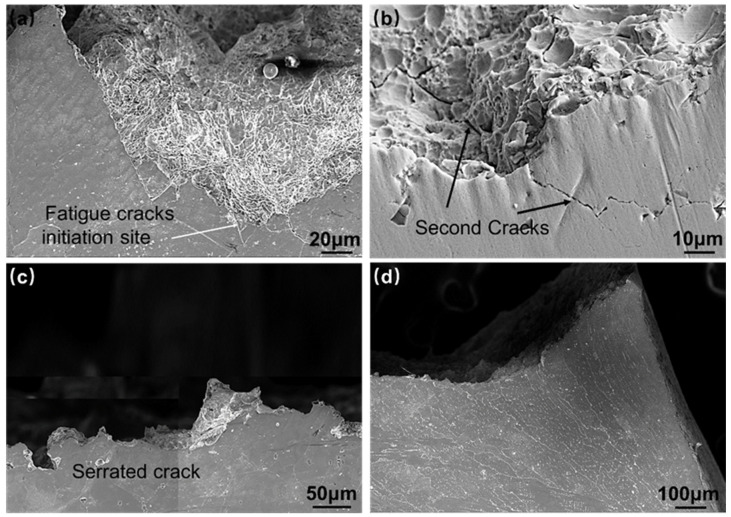
Organizational characteristics of fatigue fracture profile: (**a**) fatigue cracks initiation site; (**b**) second cracks; (**c**) serrated crack; (**d**) fatigue transient zone.

The FCG path reflects the resistance of the microstructure to the crack during the process of crack expansion, which can indicate the FCG resistance of the alloy to some extent. Figure 11 shows the cross-sectional SEM morphology of the fatigue specimen. The crack starts at the location of the surface weld toe and forms a main crack along the direction perpendicular to the tensile stress. The FCG process can be divided into three stages: the crack initiation stage, the crack extension stage, and the final failure stage. The material exhibits excellent toughness. Initially, the crack propagation occurs along the crystallographic plane close to the maximum shear stress. The direction of crack expansion gradually shifts from an angle of approximately 45° with respect to the applied stress to a direction perpendicular to the tensile stress, as illustrated in Figure 12a. Once the crack sprouts, the overall stress distribution of the specimen is no longer uniform, and there is often a large stress concentration at the crack tip, leading to an increase in crack length and a change in the expansion path. Secondary cracking occurs during the crack extension stage, as shown in Figure 12b. The front extension zigzag is larger but relatively smooth, while the later extension zigzag is smaller but has more serrated cracks, as in Figure 12c. Figure 12d shows the morphology of the ductile fracture’s instantaneous fracture zone.

**Figure 13:**
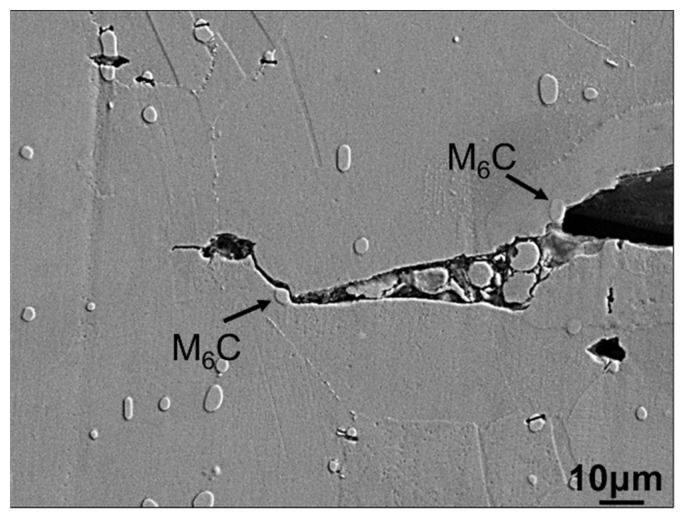
Morphology of the crack propagation path bypasses the dispersed M_6_C particles.

During the fatigue test, the specimen undergoes a gradual increase in surface temperature, leading up to failure. To capture the morphology near the crack tip, real-time monitoring utilizing a thermal imager is employed. The fatigue test is terminated before a significant rise in surface temperature occurs. SEM photographs of the cross-sectional morphology near the crack tip are obtained, as depicted in Figure 13. It was observed that the crack propagation path would bypass the dispersed M_6_C particles. He Y. et al. [33] discovered that in the case of this particular alloy, cracks tend to deflect when approaching dispersed particles, resulting in a more severe plastic closure effect and widespread crack branching. There are numerous eutectic structures present in the WM and HAZ. When the crack approaches these structures, it will cut through them but bypass tiny particles within the structure. This bypassing of numerous tiny particles leads to a significant amount of crack deflections, resulting in a higher degree of roughness-induced closure effect and retarding the crack propagation rate. This is the primary reason for the alloy’s strong resistance to crack propagation.

**Figure 14:**
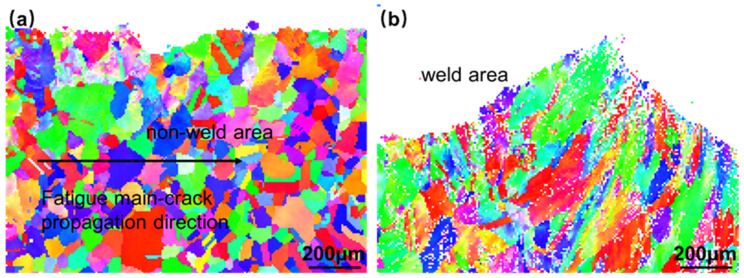
EBSD inverse pole-figures exhibiting the FCG path of Hastelloy N alloy welded joint in different locations: (**a**) the stable extension area; (**b**) the transient breaking area.

**Figure 15:**
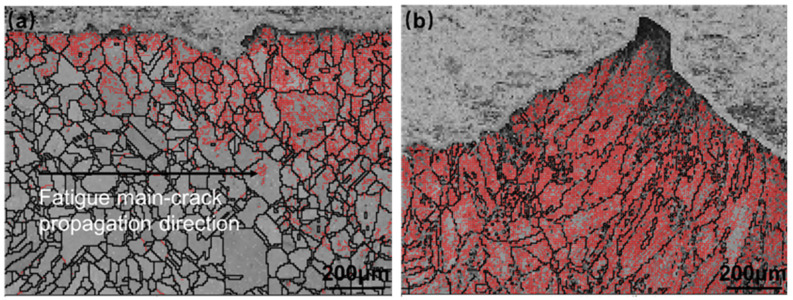
EBSD GBs maps for the FCG path of Hastelloy N alloy welded joint in different locations: (**a**) the stable extension area; (**b**) the transient breaking area.

**Figure 16:**
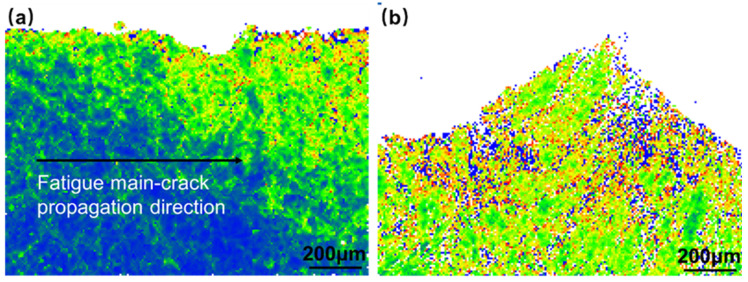
EBSD local misorientation maps for the FCG path of Hastelloy N alloy welded joint in different locations: (**a**) the stable extension area; (**b**) the transient breaking area.

Figure 14 illustrates the inverse pole figure (IPF) diagram in the Z-direction, revealing a uniform distribution of grain orientation. The non-weld area exhibits relatively fine grains, while the weld area displays coarser grains. During crack expansion, the crack propagates by crystal fracture. Figure 15a, presenting the grain boundary (GBs) diagram, demonstrates that, during stable crack propagation, low-angle grain boundaries are primarily concentrated near the crack. This suggests that the plastic deformation zone formed near the crack tip is minimal. Upon crack passage, apart from the vicinity of the crack, the dislocation density is low, indicating an elastic deformation state. Figure 15b shows the crack tip at the welded joint location, where the grain size is coarse, and there are many low-angle grain boundaries. In addition to the crack boundary, there are also many low-angle grain boundaries on the grain boundaries. Fewer low-angle grain boundaries appear inside the grains, indicating that there is a high density of dislocations at the grain boundaries in this state, which enhances stress concentration and promotes crack tip propagation. Except for the area near the crack location, there are numerous low-angle grain boundaries at an angle of about 45° along the direction of crack propagation within the grain where the crack tip is located. This indicates that there is severe dislocation accumulation at this position, and the crack tends to deflect in this direction. Figure 16 presents the kernel average misorientation (KAM) diagram, which further confirms the observations made from Figure 16. Figure 16b shows that most grains appear green, and a few regions around the GBs have blue and red color. This means that the distribution of local misorientation is highly non-uniform, indicating very high deformation at that location in the fatigue specimens. In addition, the internal stresses increase at the grain boundaries, and the intragrain stresses are relatively low compared to Figure 16a, which shows a high relative internal stress near the crack location.

## 4. Conclusions

This study investigated the microstructure, hardness, tensile strength, and fatigue properties of the GTAW welded joint of the Hastelloy N alloy. The main conclusions are given below:

The alloy primarily consists of a single austenitic phase with fine, diffusely distributed grain boundaries and intracrystalline M6C-type carbides. The highest hardness value is found near the fusion line, while the average hardness value of the WM is lower than that of the HAZ. The lowest hardness values are observed in the BM.

The fracture mode is a transgranular fracture. The carbide particles present in the material act as a strengthening phase, causing crack deflection and a strong plastic closure effect. The welded joint exhibits high resistance to crack growth and demonstrates strong fatigue resistance.

Cracking occurs in the HAZ at the weld toe. A large number of secondary cracks appear during the crack extension process. The plastic deformation zone near the crack tip is small, but a significant number of dislocations form, particularly at the grain boundaries, near specimen failure. This occurrence is primarily attributed to the reduction of the effective bearing area and the applied force exceeding the yield strength, resulting in substantial local plastic deformation.

## Figures and Tables

**Table 1:** Welding parameter.

Welding Stick	Welding Current (A)	Arc Voltage (V)	Welding Speed (mm/min)	Front Gas Flow (L/min)	Back Gas Flow (L/min)
ERNiMo-2	90	12	40	14	12

**Table 2:** EDS analysis of the phases in Hastelloy N alloy welded joint (at. %).

Elements	Ni	Mo	Cr	Fe	Cu	Ti	Si
P	68.59	15.43	8.25	4.93	2.01	0.46	0.34
Q	30.09	54.44	8.12	2.25	1.91	0.13	2.46
R	39.68	47.12	6.63	1.38	1.68	0.39	2.59
S	31.36	55.88	8.50	1.30	0.38	0.48	2.09

**Table 3:** Tensile properties.

	Tensile Stress Rm (MPa)	Yield Stress R_0.2_ (MPa)	Elongation ζ
Base metal	760	290	46
Welded joint	764	380	19

## Data Availability

Data will be made available on request.
